# A Cross-Sectional Analysis of Interventional Clinical Trials in High-Grade Glioma Therapy

**DOI:** 10.3390/life14080926

**Published:** 2024-07-24

**Authors:** Angelo Angione, Jonathan Patterson, Ebrar Akca, Jessica Xu, Emily Xu, Vanessa Raab, Omar Elghawy, Adam A. Barsouk, Jonathan H. Sussman

**Affiliations:** 1 Department of Neurosurgery, Hospital of the University of Pennsylvania, Philadelphia, PA 19104, USA; 2 Icahn School of Medicine at Mount Sinai, New York, NY 10029, USA; 3 Biomedical Sciences Training Program, Case Western Reserve University School of Medicine, Cleveland, OH 44106, USA; 4 Department of Medicine, Hospital of the University of Pennsylvania, Philadelphia, PA 19104, USA; 5 Graduate Group in Genomics and Computational Biology, Perelman School of Medicine, University of Pennsylvania, Philadelphia, PA 19104, USA

**Keywords:** high-grade glioma, glioblastoma, clinical trial demographics, health disparities, socioeconomic status, rural healthcare access

## Abstract

High-grade glioma is the most frequent and lethal primary tumor of the central nervous system. Despite advances in surgical, pharmacological, and cell-directed therapies, there have been no updates to the standard of care in over a decade. This cross-sectional study analyzes patient and trial data from 201 interventional trials completed between 2010 and 2023, encompassing 18,563 participants. Although we found that all trials reported participant age and sex, only 52% of trials reported participant demographics, resulting in 51% of total participant demographics being unreported. The majority of studies did not report ethnicity, with approximately 60% of participants unreported. Additionally, males were significantly underrepresented in trials, comprising 60% of participants despite representing 75% of glioblastoma patients. Improved demographic reporting has been observed since 2011; however, it is inconsistent. Furthermore, we cataloged the geographic diversity of trials across the United States and found significant coverage deserts in relatively rural, but highly affected, areas such as Montana and Maine. We found a wider distribution of trials in both urban and wealthier regions, which indicates extensive coverage gaps and decreased access to participation for patients of a lower socioeconomic status.

## 1. Introduction

Gliomas comprise the most common primary malignant tumors of the central nervous system (CNS). Of these, glioblastoma (GBM) is the most common and aggressive. In the United States, GBM has an average annual incidence of 12,652 new cases and comprises 14.2% of all CNS tumors. It has a 6.7% median 5-year survival rate [1]. GBM, and all high-grade gliomas, currently remain incurable.

Adult gliomas are classified as high- or low-grade according to the World Health Organization (WHO) 2021 classification. WHO grade 1 and 2 tumors are considered low-grade and are potentially curable by surgical resection. These slower-growing tumors, however, often progress to become high-grade gliomas (WHO grade 3 and 4) [2]. High-grade gliomas are aggressive, infiltrative, and immunologically cold, presenting significant therapeutic challenges. The current standard of care has existed, relatively unchanged, for over a decade, without appreciable increases in overall survival [3]. Standard multimodal therapy includes magnetic resonance imaging (MRI)-guided maximal safe surgical resection, followed by radiotherapy and adjuvant or concurrent chemotherapy. This has yielded an overall median survival of 14.6 months [3,4,5]. In addition to this strategy, dexamethasone or other steroids are utilized as palliative interventions, and the anti-angiogenic agent bevacizumab has been FDA-approved as an adjuvant treatment [5]. Other therapeutic options include intraoperative or oral nitrosoureas (i.e., lomustine, carmustine) and tumor-treating fields [6], although these have only modestly affected patient outcomes. The highly diffuse and infiltrative nature of GBM limits the curative potential of surgical resection. Major challenges in the development of pharmacological agents include poor blood–brain barrier penetrance and the chemoresistance and radioresistance of glioma neoplastic cells, resulting in high recurrence rates. These challenges necessitate thorough investigation of novel treatment modalities and extensive integration of basic science advancements into the clinical sphere.

In this study, we sought to characterize the current landscape of interventional clinical trials treating adult high-grade gliomas cataloged by https://www.clinicaltrials.gov/ (accessed on 5 May 2024). ClinicalTrials.gov functions as an essential database for researchers and is the first line of information across hundreds of thousands of clinical studies. We striated trials by participant reporting and recruitment, intervention, and geographic diversity to identify areas of weakness in patient accessibility or research reporting.

## 2. Materials and Methods

This cross-sectional study utilized data collected from interventional adult high-grade glioma clinical trials of all stages completed between 1 April 2010 and 1 April 2023 that were registered and reported to ClinicalTrials.gov. The terms “Glioma”, “GBM”, and “High Grade Glioma” were searched as “Condition/Disease” query items on 23 April 2024 in the ClinicalTrials.gov database. The results were further refined to interventional studies completed between 1 April 2010 and 1 April 2023, enrolling adults (ages 18 and older) and excluding child participants. The returned studies were further filtered to exclude trials with unreported results and studies with goals that do not directly attempt to treat high-grade gliomas. Incidence and mortality data were collected from the National Cancer Institute’s Surveillance, Epidemiology, and End Results (SEER) program [7]. Only trial data from United States participants were used for demographic analysis, although trials conducted in part at international sites are also reported in this database.

As this study utilized publicly available deidentified data and did not involve human participants, it did not require institutional review or approval by an institutional ethics committee or review board. This study followed the Strengthening the Reporting of Observational Studies in Epidemiology (STROBE) reporting guideline.

### 2.1. Data Collection

We collected descriptive data of the trials, including funding sources, costs, partner institutions, and trial site information. We additionally collected demographic data of participants, including race/ethnicity, sex, and age, as reported by each included trial on its ClinicalTrials.gov study record. Race/ethnicity categorization that is consistent with the Office of Management and Budget Standards for the Classification of Federal Data on Race and Ethnicity (race OMB) [8] was noted. However, not all trials were reported in this manner. Trials were stratified by investigational modality (i.e., drug, radiation, surgical procedure, etc.), year, and trial phase. Combined Phase 1/2 trials were encoded as Phase 1 trials unless they contained more than 50 participants, which were encoded as Phase 2.

United States demographic data for comparison to the general population was collected from the US Census American Community Survey, 2016 [9,10]. A sampling error of ±0.1% or less was generally reported.

### 2.2. Race and Ethnicity Reporting

The percentage of trials reporting race and ethnicity demographics was computed for each year. The count of trials reporting race and ethnicity was divided by the total number of trials for each respective year.

The total number of participants in clinical trials per year was calculated by the “Results Submitted Year” data field. Percentages were calculated for each racial and ethnic category, including White, Black or African American, Asian, American Indian or Alaska Native, Native Hawaiian or Pacific Islander, individuals identifying with multiple races (more than one race; mixed race), those with Unknown or Other category race, and Hispanic or Latino individuals. The percentages were calculated by dividing the count of participants in each category by the total number of participants for the respective year.

### 2.3. Trial Stratification and Analysis

Trials with 20 or fewer recruiting locations were then stratified into “Public”-, “Private”-, or “Mixed”-institution trials. “Public Institutions” were qualified as Veteran’s Affairs, public-university-associated hospitals (e.g., University of Pittsburg Medical Center), or the NIH Clinical Center. All other institutions were qualified as “Private Institutions”. Trials involving both a “Public” and a “Private” institution were classified as “Mixed”. Trials containing more than 20 US recruitment centers were designated as “large multicenter trials” and not stratified by institution type. Large multicenter trials and trials not disclosing their recruitment centers were excluded from this stratification.

Trials were classified according to the year the results were submitted. Data from trials that had international testing sites were excluded from demographic analyses. The percentage of trials reporting race and ethnicity was computed for each year and trial type (public, private, or mixed). The percentage of trials reporting race by OMB standards was also calculated. The percentage of trials reporting race and ethnicity was computed for each year and funder type (NIH, industry, network, or other). “Other”, as defined by ClinicalTrials.gov, includes individuals, universities, and community-based organizations. The percentage of trials reporting race and ethnicity was computed for each year and trial phase. Incidence rate [7], count of sites, count of trials, and median annual income [9,10] per state were collected as previously reported.

### 2.4. Statistical Analysis

All statistical analyses were performed in R (4.3.1) (R Foundation for Statistical Computing, Vienna, Austria). Spearman’s rank correlation coefficients were used to identify correlations. Two-proportion z-tests were used to compare percentage data. For demographic data, the United States population was used as the denominator. *p*-values < 0.05 were considered statistically significant. All *p*-values and Spearman’s Rho values were reported as numerical values within tables and figures as appropriate.

## 3. Results

### 3.1. Trial Characteristics

**Figure 1:**
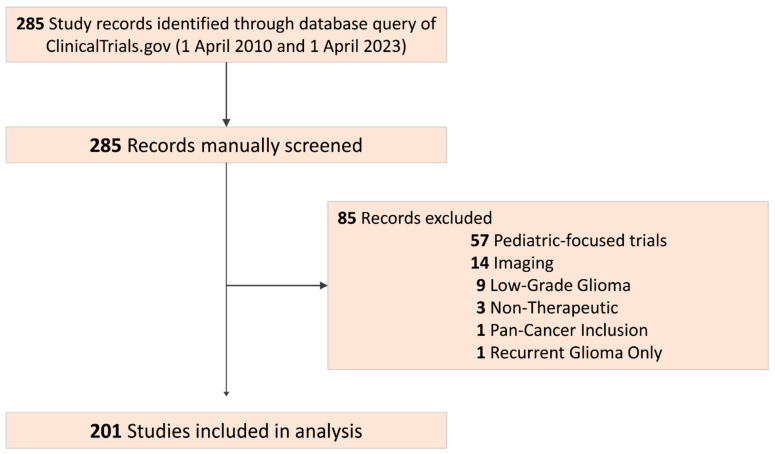
Overview of search strategy for identification of relevant clinical trials.

Our query returned 285 total studies. We then manually filtered out studies that did not address the treatment of adult high-grade gliomas, including studies on pediatric patients, and those involving imaging modalities or caregiver quality of life (Figure 1). This resulted in a total of 201 trials which were subsequently screened for reported participant data, which was then pooled (Table 1, Supplemental Tables S1 and S2). We identified 2 Pilot studies (1%), 3 studies designated Early Phase 1 (1.5%), 30 Phase 1 (14.9%), 153 Phase 2 (76.1%), and 13 Phase 3 (6.5%). Of the included trials, 106 were either randomized or unrandomized in design (53; 26.3% each) and the remaining 96 (47.8%) were uncategorized. While all the included trials reported the age and sex of participants, about half of the trials reported race (105; 52.2%). We identified that only 84% of trials reporting race followed the OMB reporting standards (88; 43.8% of all trials). Ethnicity was the lowest reported demographic characteristic, with only 78 (38.8%) trials reporting.

Trials were organized by year of publication and stratified by the type of investigational intervention: drugs (83; 41.3%), interventional radiation (44; 21.9%), surgery (23; 11.4%), biologics (27; 13.4%), and other interventions (36, 17.9%). These groups were then reclassified to differentiate trials utilizing multiple investigational agents of the same or different types (150; 74.6%). For example, bevacizumab, temozolomide, and radiosurgery would be classified as two drugs and interventional radiation. Multimodal investigational strategies (91; 45.2%) utilize two or more distinct types of interventional agents, such as avelumab (biologic) and hypofractionated radiation therapy (interventional radiation).

### 3.2. Race and Ethnicity

We pooled participants within each OMB/US Census-identified demographic group reported for all 105 studies reporting race. This resulted in demographic data reported for 9098 of 18,563 (49%) participants (Table 2). We then compared the proportions of participants within each group with the proportion of Americans as identified by the US Census American Community Survey. Independent of the year, we identified White participants (86.7%) to be overrepresented (*p* < 0.0001). American Indian or Alaska Native (0.6%) and Native Hawaiian or Pacific Islander (0.2%) participants were proportionally represented. Black or African American (3.1%, *p* < 0.0001), Asian (4.6; *p* = 0.0012), and mixed race (0.2%; *p* < 0.0001) participants were found to be significantly underrepresented within trials reporting demographics. We found that the number of participants reported to be of unknown race or unable to be categorized within these groups (4.5%) were underrepresented compared to the American Community Survey (*p* = 0.010). Ethnicity was reported for 6702 (36.1%) participants in 78 (38.8%) trials. We found Hispanic or Latino-origin participants of all races (386; 5.6%) to be significantly underrepresented compared to the American average (*p* < 0.0001).

**Figure 2:**
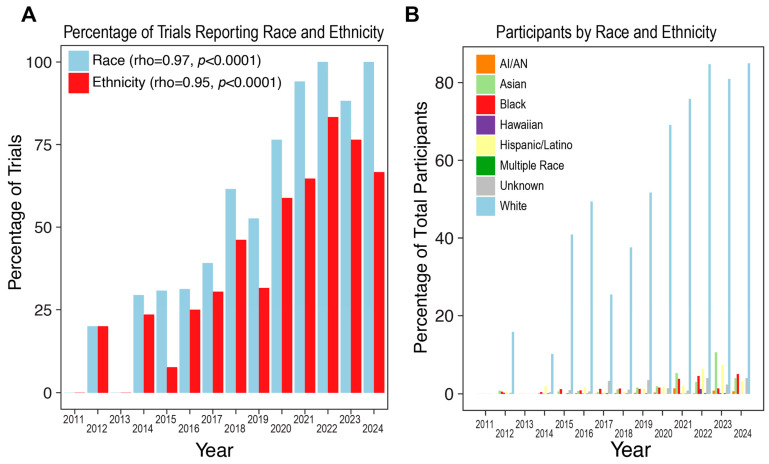
Race and ethnicity in HGG clinical trials. (**A**) Percentage of trials reporting race and ethnicity published between 2011 and 2024. Significance in longitudinal trends was calculated using Spearman’s correlation coefficient. (**B**) Clinical trial participants by race and ethnicity category. AI/AN, American Indian/Alaska Native.

Next, we grouped trials according to the year of final reporting and cataloged the proportion of participants within each racial and ethnic group reported for all trials. Here, we identified a significant positive reporting trend (Figure 2A). Additionally, we identified the reported relative enrollment of the underrepresented groups to have increased yearly (Figure 2B).

**Figure 3:**
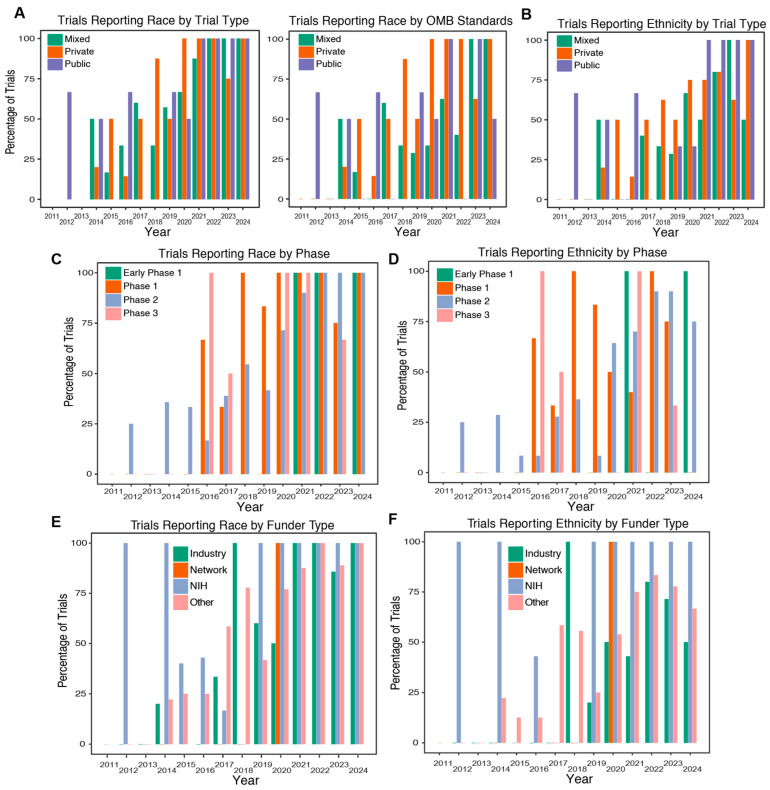
Trial demographic reporting by trial type. (**A**) Percent of reporting race by trial type (mixed, private, public) including any race reporting (left) and race reporting by OMB standards (right). (**B**) Percent of trials reporting ethnicity by trial type. (**C**,**D**) Percent of trials reporting race or ethnicity (**D**) by trial phase (Early Phase 1, Phase 1, Phase 2, Phase 3). (**E**,**F**) Percent of trials reporting race (**E**) or (**F**) ethnicity by funder type (industry, network, NIH, other). Data are shown for the years 2011–2024.

We classified trials according to their recruiting institutions. Participants were pooled, and we found trials performed by public institutions to be the most consistent in reporting participant race, followed by mixed-type institutions (Figure 3A). Private institutions, while having the lowest overall percentage for reporting race, showed an upward trend over time, with almost 100% reporting race between 2020 and 2024 (Figure 3A). We analyzed these data against the OMB reporting standard and similarly found public institutions to report race most consistently overall, followed by private institutions. Private institutions reported by OMB standards 100% of the time beginning in 2020 (Figure 3A). Mixed-type institutions showed the lowest adoption of OMB reporting standards but achieved 100% reporting in 2023 and 2024 (Figure 3A). When we analyzed ethnicity reporting by trial type, we found private institutions to demonstrate an upward reporting trend (Figure 3B). We identified public institutions to have the best reporting, and the only group to achieve 100% reporting in more than one year, between 2021 and 2024 (Figure 3B).

Next, we categorized trials by phase, including Pilot, Early Phase 1, Phase 1, Phase 2, and Phase 3, and analyzed their reporting trends as interventions approaching FDA approval. We identified Phase 2 trials to be the most consistent reporters overall, followed by Phase 1 and Phase 3 trials. Early Phase 1 trials followed an “all or none” reporting trend, with 100% reporting in 2021, 2022, and 2024, and 0% reporting in all other years (Figure 3C). Ethnicity reporting was also observed to be most robust in Phase 2 trials, with a similarly strong upward trend, but never reaching 100% reporting in any single year (Figure 3D).

### 3.3. Sex

Male participants were observed to be overrepresented within all trials (*p* < 0.0001) when compared to the overall American population, with female participants conversely underrepresented (Table 2). However, when compared to the incidence of glioblastoma, we found males to be significantly underrepresented in trials (*p* < 0.0001) (Table 2). However, the magnitude of this difference is small and does not necessarily represent a clinically meaningful imbalance in participants.

### 3.4. Funding Sources

We identified the majority of studies were funded privately (“Other”, 120; 59.7%), followed by industry-funded (40; 19.9%) and NIH-funded (36; 17.9%) studies (Table 1). We grouped the studies according to the type of institution that performed each trial. The majority were completed by private academic and business ventures (72; 35.8%), followed by studies conducted by both public and private entities (“Mixed”, 54; 26.9%). We then compared trial reporting of enrolled participant demographics stratified by their funding source. We found the overall most consistent reporters of both race and ethnicity to be NIH-funded across all years, followed by private/third-party-funded trials (“Other”) with an upward trend over time (Figure 3E,F). Industry-funded trials also demonstrated a strong upward trend in race reporting, but not ethnicity reporting, over time. Network-funded trials showed 100% reporting, although the sample size was relatively small (five, 2.5%).

### 3.5. Geographic Diversity

**Figure 4:**
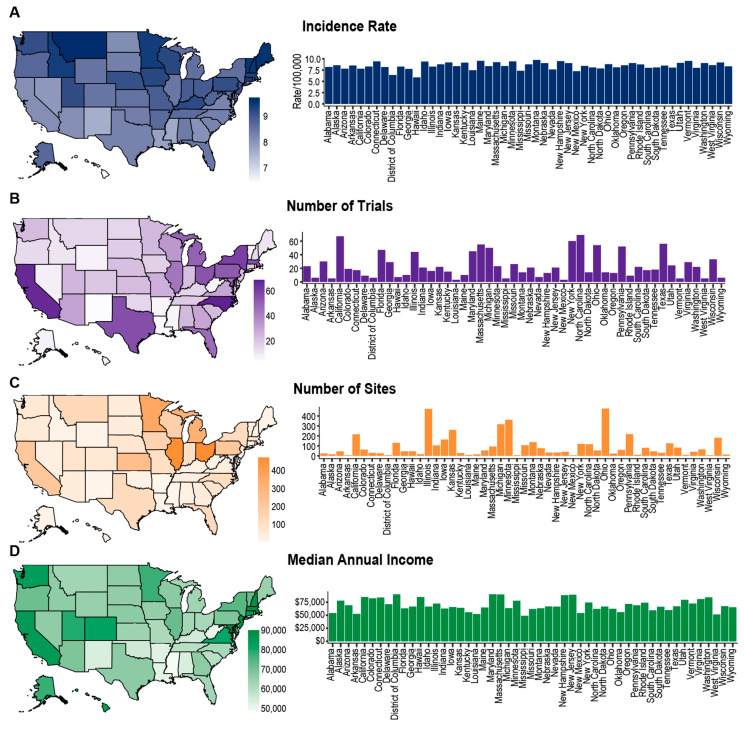
Geographic characteristics of high-grade glioma clinical trials across the United States. (**A**) Incidence rate of high-grade glioma by state, shown as rate per 100,000 individuals. (**B**) Number of independent clinical trials conducted in each US state. (**C**) Number of clinical trial sites in each state. (**D**) Median annual household income (US dollars) in each state.

We analyzed the geographic distribution of trials and trial sites throughout the United States. Utilizing the SEER database, we identified a trend towards a higher incidence of high-grade glioma in northern states (Figure 4A) with New England, Minnesota, Idaho, and Montana identified as regions of particularly high incidence (Figure 4A). The majority of trials utilized a multi-site design, and approximately half utilized sites in multiple US states (100, 49.8%) (Table 1). We identified trial “hotspots” in California, Massachusetts, New York, North Carolina, and Texas (Figure 4B). We found most recruitment sites to be located within Illinois, Ohio, and Massachusetts (Figure 4C). Notably, the locations hosting the largest number of trials or sites, with the exception of Massachusetts, are outside the highest-incidence states. The states hosting the greatest number of trials were observed to coincide with the locations of the largest academic cancer centers in the country, such as the MD Anderson Cancer Center and Duke Medical Center. However, the largest number of recruitment sites were concentrated within the Midwest region.

### 3.6. Socioeconomic Status

Utilizing US Census data, we tabulated the median average household income of each state (Figure 4D). We identified West Virginia, Mississippi, Alabama, New Mexico, Arkansas, and Oklahoma to represent the poorest states in the country. We observed a notable absence of clinical trial recruitment within these states (Figure 4B,C). Additionally, we observed a similar paucity within Nevada, Wyoming, Vermont, Maine, Louisiana, Alabama, and Idaho. Our data suggest West Virginia and New Mexico to be locations of particularly high need and notably low accessibility. These results indicate an enrichment of clinical trials towards locations of higher average socioeconomic status, such as California and New York. However, the presence of large academic institutions within urban centers improves accessibility. This suggests that while the average socioeconomic status of a given state may play a role in trial distribution, the urban sprawl within the state may contribute to these trends, with more rural locations remaining more underserved.

## 4. Discussion

Glioblastoma, the most aggressive and common primary malignancy of the brain, remains incurable despite significant efforts to advance care from both clinical and basic science perspectives. Additionally, this disease manifests asymmetrically, requiring robust clinical investigation within patient cohorts that are representative of these disparities [11,12]. In this study, we sought to characterize the current clinical investigational landscape of high-grade glioma within the United States and identify areas of weakness in reporting and recruitment metrics.

Adequate representation of patients within clinical research cohorts has been a well-documented concern affecting research outcomes and treatment modalities [13,14]. Additionally, in the case of diseases such as glioblastoma, which afflicts patients disproportionately [12], clinical trial inclusion and availability function to minimize disparities among outcomes [15,16]. Within our study, we demonstrate how, over time, racial and ethnic inclusion within trials has improved. However, despite these improvements, there remains significant room for growth, as some minority groups [17] remain underrepresented in clinical trials. Interestingly, it has been reported that participation of these groups has grown substantially across all clinical trials [17]. As other groups have reported [16,17,18,19], transparency of enrollment remains poor systemically. As recently described [19], further investigation and increased stringency on behalf of the Food and Drug Administration (FDA) are necessary for accurate and robust interrogation.

Secondly, our study aimed to identify trends between access, need, and geographic distribution of glioma trials, an avenue not thoroughly explored in almost a decade [20,21]. We found that high-grade glioma incidence does not correlate with the number of trials or sites per state. The number of trials did coincide with the location of large academic cancer centers. While this trend is not surprising, it poses a major barrier to treatment access, particularly for patients who may be unable to seek treatment at these large centers.

Most strikingly, however, was the correlation between socioeconomic status and the presence and accessibility of investigational care. We identified only three trials containing sites in Louisiana and New Mexico, and five in Mississippi, Vermont, Arkansas, and West Virginia. All of these states also hosted less than 10 trial sites. The relatively underserved and rural nature of these regions additionally suggests greater barriers to care than just the presence of clinical trials, as decreased availability of care has been well documented, both in the United States and abroad [22,23]. It has been well documented that patients within these medically underserved areas face inferior screening and diagnosis, and therefore, worse prognoses [24]. However, no trials provided conclusive data of the residence of their participants beyond recruitment location. This implies that participants were enrolled from a relatively local radius of the recruitment center.

Moreover, this study reveals specific ways in which trial demographic reporting could be improved. We found that many studies substituted their own scales of age, race, or other participant characteristics and did not adhere to the OMB guidelines. This hampered the ability to compare characteristics across trials accurately. Additionally, patient characteristics are often not easily accessible on ClinicalTrials.gov, with key demographic information only present within trial appendices and supplemental documentation. This creates a barrier to thoroughly cataloging participant metrics across thousands of clinical trials. Clinical trial recruitment is crucial to improve the standard of care for fatal diseases such as glioblastoma for all patients. By standardizing and promoting demographic reporting, critical gaps in accessibility to investigational care can become apparent and addressed. While this study contains broad-reaching aims, it is limited in focus to the state level, and further granularity in patient metrics is needed in future studies. For example, participants in trials recruited at locations close to state borders, such as those within New York City, would recruit participants residing in at least one adjacent state. This limits the resolution of our analyses to the presumption that a substantial majority of participants reside within the state of a given trial’s registration. Even if these metadata are collected, they are not reported by ClinicalTrials.gov. We were additionally limited in our analysis of participant age and race due to inconsistency inherent within the data. There has been an upward trend in OMB reporting adherence; however, the 56.2% of included trials that did not adhere to these reporting standards limit our capacity to make longitudinal comparisons. Finally, demographic data are provided from these studies following their completion and do not account for patient enrollment over time. This limits the evaluation of longitudinal changes in demographic enrollment within groups, which may change over the extended time period of the clinical trials. Further research is necessary to fully understand the longitudinal trends within and between glioma clinical trials.

## Figures and Tables

**Table 1:** Characteristics of high-grade glioma clinical trials registered in www.ClinicalTrials.gov.

Characteristic	Number of Trials (% of All Trials), n = 201
Randomized	53 (26.3)
Unrandomized	53 (26.3)
Single Testing Site	94 (46.8)
Multiple Testing Sites	107 (53.2)
Testing Sites in Multiple States	100 (49.8)
Multiple Testing Sites Across Single State	34 (16.9)
Includes International Testing Sites	33 (16.4)
Private Investigational Institution	72 (35.8)
Public Investigational Institution	31 (15.4)
Mixed Investigational Institution	54 (26.9)
Investigational Institution Not Reported	44 (21.9)
Reports Race	105 (52.2)
Race Reported by OMB Standard (Of Trials Reporting Race)	88 (43.8)
Reports Ethnicity	78 (38.8)
Reports Sex	201 (100)
Reports Age	201 (100)
Intervention Type: Drug	83 (41.3)
Intervention Type: Radiation	44 (21.9)
Intervention Type: Surgical	23 (11.4)
Intervention Type: Biologic	27 (13.4)
Intervention Type: Other	36 (17.9)
Utilizes Multiple Investigational Agents	150 (74.6)
Utilizes a Multimodal Interventional Strategy	91 (45.2)
Phase Pilot	2 (1)
Phase Early 1	3 (1.5)
Phase 1	30 (14.9)
Phase 2	153 (76.1)
Phase 3	13 (6.5)
NIH-Funded	36 (17.9)
Industry-Funded	40 (19.9)
Network-Funded	5 (2.5)
Other Funder	120 (59.7)

**Table 2:** Participant characteristics by race/ethnicity and sex in registered high-grade glioma clinical trials and the US population.

Characteristic	Number of Participants (% of Reported) n = 18,563	% of US Adult Population in 2016 n = 323,100,000	*p*-Value
Male	11,029 (59.5)	49.2 (Incidence: 4/100,000)	**<0.0001**
Female	7514 (40.5)	50.8 (Incidence: 2.5/100,000)	**<0.0001**
Sex Not Reported	20 (0.1)	N/A	**N/A**
White	7889 (86.7)	72.6	**<0.0001**
Black or African American	282 (3.1)	12.7	**<0.0001**
Asian	421 (4.6)	5.4	**0.0012**
American Indian or Alaska Native	57 (0.6)	0.8	0.069
Native Hawaiian or Pacific Islander	22 (0.2)	0.2	0.40
More Than One Race	17 (0.2)	3.2	**<0.0001**
Other/Unknown	410 (4.5)	5.1	0.010
Race Not Reported	9466 (51.0)	N/A	N/A
Hispanic or Latino	386 (5.8)	17.8	**<0.0001**
Ethnicity Not Reported	11,862 (63.9)	N/A	N/A

Significance was assessed using a 2-proportion z-test. Bold indicates statistical significance (*p* < 0.05). N/A, not applicable.

## Data Availability

Any additional data and code used to generate the analysis and figures in the current study are available from the corresponding author upon reasonable request.

## Supplementary Materials

The following supporting information can be downloaded at https://www.mdpi.com/article/10.3390/life14080926/s1: Table S1: Complete clinical trials included in this study with demographic and institutional information. Table S2: Additional clinical trial information, including summaries of the studies and interventions tested.
